# Sex Differences in the Epigenome: A Cause or Consequence of Sexual Differentiation of the Brain?

**DOI:** 10.3390/genes10060432

**Published:** 2019-06-07

**Authors:** Bruno Gegenhuber, Jessica Tollkuhn

**Affiliations:** 1 Cold Spring Harbor Laboratory, Cold Spring Harbor, NY 11724, USA; gegenhu@cshl.edu; 2 Watson School of Biological Sciences, Cold Spring Harbor Laboratory, Cold Spring Harbor, NY 11724, USA

**Keywords:** sex differences, epigenetics, estrogen, neurodevelopment, gene regulation

## Abstract

Females and males display differences in neural activity patterns, behavioral responses, and incidence of psychiatric and neurological diseases. Sex differences in the brain appear throughout the animal kingdom and are largely a consequence of the physiological requirements necessary for the distinct roles of the two sexes in reproduction. As with the rest of the body, gonadal steroid hormones act to specify and regulate many of these differences. It is thought that transient hormonal signaling during brain development gives rise to persistent sex differences in gene expression via an epigenetic mechanism, leading to divergent neurodevelopmental trajectories that may underlie sex differences in disease susceptibility. However, few genes with a persistent sex difference in expression have been identified, and only a handful of studies have employed genome-wide approaches to assess sex differences in epigenomic modifications. To date, there are no confirmed examples of gene regulatory elements that direct sex differences in gene expression in the brain. Here, we review foundational studies in this field, describe transcriptional mechanisms that could act downstream of hormone receptors in the brain, and suggest future approaches for identification and validation of sex-typical gene programs. We propose that sexual differentiation of the brain involves self-perpetuating transcriptional states that canalize sex-specific development.

## 1. Introduction

“In the study of development we are interested not only in the final state to which the system arrives, but also in the course by which it gets there” C.H. Waddington, 1957.

As described in detail [1,2,3], the definition of epigenetics has evolved over time, leading to a relaxed usage of the term, particularly in popular culture (see “From epigenetic landscapes to epigenetic pancakes” at http://blogs.nottingham.ac.uk/makingsciencepublic/). In the study of sex differences—and in the field of neuroepigenetics in general—“epigenetic” refers to covalent DNA or histone modifications that exist “over” DNA and influence gene expression. Frequently, differences in the epigenome, primarily in DNA methylation, are assumed to be causal for phenotypic differences in gene expression, disease incidence, or behavior. However, when Waddington coined the term “epigenetics” in 1942 (republished as Reference [4]), ten years prior to the Hershey-Chase experiments [5], he had no concept of the physical nature of a gene or what we now call the “epigenome”. Rather, he intended to symbolize a developmental process through an “epigenetic landscape” [6], in which each valley represents the segregation of “developmental competence”, or the cell fate decisions made throughout embryogenesis. In Waddington’s landscape, “equilibrium is not centered on a static state, but rather on a direction or pathway of change” [7]. This concept is represented by the term “homeorhesis” (*rhesis* meaning flow) to distinguish it from the more widely-used “homeostasis”. To date, however, most research has focused on identifying epigenomic endpoints rather than elucidating processes that give rise to such endpoints. Throughout this article, we consider sexual differentiation of the brain as a developmental progression. We suggest that rather than focusing on the epigenomic signature that is a consequence of cell fate decisions, researchers should identify the regulatory mechanisms of transcription factors that establish and/or maintain this signature, as this is crucial for understanding the origin of sex differences in gene expression. 

## 2. Hormone Signaling at Birth Defines Sex Differences in Brain Function

Sex differences in the brain arise from contributions of sex chromosomes and gonadal steroid hormones. The classic Organization and Activation hypothesis, first articulated sixty years ago, states that hormone signaling in early life specifies sex differences in the brain, which are subsequently activated by adult hormones to produce sex-typical reproductive and territorial behaviors [8,9]. At birth, male mice and rats experience a dramatic increase in circulating testosterone that is converted to estradiol directly in the brain by aromatase, a P450 enzyme [10,11]. Estradiol binds its canonical receptors estrogen receptor alpha and beta (ERα/β), which are nuclear receptor transcription factors that are recruited to DNA in response to ligand binding [12]. Estrogens can also act rapidly at the cell membrane to increase neuronal firing [13,14,15,16], potentially initiating activity-dependent transcriptional programs that differ from those directed by nuclear ERα [17]. ERα is thought to be the master regulator of sexual differentiation of the brain. Genetic deletion of this receptor attenuates male-typical sexual and territorial behaviors and feminizes the expression of ERβ and androgen receptor (AR), the receptor for testosterone [18,19,20,21,22]. ERβ also plays critical roles in sexual receptivity, fertility, lactation, and other aspects of female physiology. 

Neonatal exposure to estradiol alters the developmental trajectory of the brain, affecting cell number, differentiation, and wiring days after the hormone surge has subsided [23,24]. In males, estradiol induces neurite outgrowth and promotes cell survival in some brain areas, such as the medial amygdala (MeA) and bed nucleus of the stria terminalis (BNST) [25,26], while simultaneously initiating apoptosis in the anteroventral periventricular (AVPV) hypothalamus, a region that regulates ovulation in females [25,27]. Events such as these contribute to sex differences in the function of innate behavioral circuitry. For instance, males and females engage in different mating routines, and only males urine-mark their territory and aggressively defend it, although lactating females will attack intruding conspecifics to defend their offspring [28,29,30,31]. There are also extensive sex differences in stress responses and motivated behaviors [32,33,34]. During estrus, females are sexually receptive to males. Yet if females receive estradiol during the first week of life, adult estrus elicits aggression toward males instead of receptivity [26]. How do females “remember” an estradiol treatment weeks after it has subsided? 

**Figure 1:**
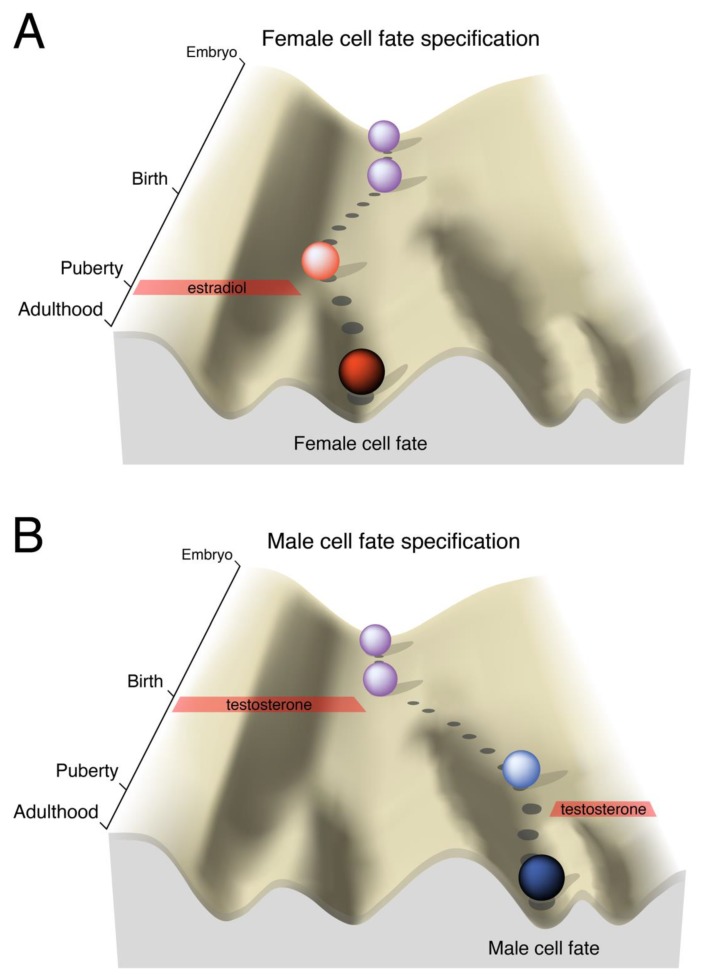
Developmental landscapes of brain cell fate specification. In Waddington’s description of the epigenetic landscape [6,7], the ball rolling down the hill represents the developmental trajectory of an embryo: The landscape itself is pre-specified by the genetic makeup of the organism, but the chosen path can be altered by extrinsic events. As development progresses, the valley slopes steepen, representing the inevitable canalization of a genetic program as cells differentiate and their plasticity is restricted. Near birth, the rodent brain is bipotential and can easily progress to a female or male fate. **A)** The brain, like the gonads, develops by default as female. **B)** In males, testosterone is converted to estradiol in the brain and activates a gene expression program that irreversibly pushes the brain towards a male fate. In both sexes, puberty is an additional sensitive period when sexually differentiated circuitry is acted on by sex-specific hormonal profiles.

The field has long assumed that estradiol directs sexual differentiation of the brain via an epigenetic mechanism, whereby transient exposure to estradiol during a neonatal critical period irreversibly modifies the chromatin state of gene regulatory elements [35,36,37,38,39,40,41], schematized in Figure 1. This process could result in either a constitutive sex difference in gene expression or a sex-specific transcriptional response to a later signaling cue. Much has been written on this subject, for recent reviews see References [42,43,44,45], and transcriptomic analyses in model organisms and humans have identified genes differentially expressed between the brains of the two sexes [17]. However, only a handful of genes have been found to be regulated by neonatal estradiol in the developing male brain, and there are still no examples of sexually dimorphic chromatin states at specific loci that are causal for sex differences in gene expression. In addition, the gene regulatory strategies employed by hormone receptors in the brain remain obscure, as ERα and AR have primarily been studied in human cancer cell lines. 

Here we integrate recent findings on epigenetic regulation of gene expression in the brain and nuclear receptor biology to propose a transcriptional model for how neonatal estradiol could specify the development of a male-typical brain. Notably, although Figure 1 depicts a binary choice between two outcomes–female or male–there are multiple sexual differentiation events in the brain. Due to intrinsic transcriptional and epigenetic heterogeneity, different cell types may adopt unique cell fates in response to neonatal estradiol. The aggregation of such cell fates could then produce a spectrum of sexually differentiated phenotypic states. Or, as Waddington said: “The alternative between maleness and femaleness is not so definite… the intersexual condition is no more a case of sharp alternative than is pituitary dwarfism. It becomes merely one of the numerous cases in which the normal well-defined alternatives are disrupted by changes in the genotypic system on which they are based” [6].

## 3. Regulation of Gene Expression in the Brain

Mammalian gene transcription is directed by transcription factors and influenced by DNA methylation, post-translational modifications (PTMs) to histone proteins, and chromatin organization [46,47,48]. While hormone receptors have been shown to regulate these properties in cell culture models, particularly breast (ERα) and prostate (AR) cancers, few studies have examined how they are regulated in the brain. Moreover, the longevity and renewal of such modifications in post-mitotic neurons remain unclear. In this section, we discuss the mechanisms by which hormone receptors regulate the epigenome and their potential role in the epigenetic maintenance of sex differences in the brain. We review studies on sex differences in the neuronal epigenome in the context of recent findings on the nature of epigenetic regulation of gene expression in the brain. Although there are other factors that contribute to sex differences in the brain, here we focus on ERα, as this receptor is the master regulator of sexual differentiation of the rodent brain, and its role in gene regulation has been extensively studied [17]. 

### 3.1. DNA Methylation

The predominant mechanism associated with the epigenetic regulation of gene expression is DNA cytosine methylation (5mC). This modification is relatively stable, correlates with gene repression or activation depending on its genomic location, and is necessary for development, as revealed by mouse genetics [49]. It can maintain genomic imprinting [50], silence transposons [51], and is required for X-inactivation [52]. In the brain, de novo DNA methylation by DNA methyltransferases (DNMTs) is required for learning and memory [53], and there are dynamic changes in DNA methylation in response to neural activity [54,55,56,57]. The mechanism of DNA demethylation in post-mitotic cells was a mystery for years, as no vertebrate homologs to known plant DNA demethylases had been identified [58]. Then in 2009, it was simultaneously published that DNA in the brain contains hydroxymethylated cytosines (5hmC) [59], and that the hydroxylation of 5mC is regulated by the ten-eleven translocation (Tet) family of enzymes, which initiate a cascade that leads to base-excision repair [60]. Further characterization of this pathway led to extensive exploration of stimuli-induced DNA demethylation in the brain [61,62].

Whole genome bisulfite sequencing (WGBS) of neurons has revealed enrichment of DNA methylation on cytosine residues that are not followed by a guanine (denoted as mCH), and that the most common alternate base is an adenine (mCA) [63]. This modification is found in other tissues, but it increases in the brains of mice and humans during postnatal development, concomitant with synaptogenesis and experience-dependent neural activity [63,64,65]. In contrast, mCG patterns are established prenatally and appear stable over time, although it is likely that alterations in mCG within specific circuits or cell types would not be detected in analyses of bulk tissue. WGBS in genetically-defined neuronal types combined with ChIP-seq analysis of Dnmt3a recruitment during postnatal development revealed that mCA is deposited by Dnmt3a over the gene bodies to maintain cell-type specific gene repression [65]. Collectively, this recent work on mCA suggests that this type of DNA methylation plays a crucial role in refining cell identity as neuronal connectivity is established. 

Can perinatal estradiol directly alter DNA methylation state and the expression pattern of associated genes? Reports of sex differences in DNA methylation in the brain have assessed either individual methylated cytosines at promoters [66,67,68] or reported percent methylation across the entire genome [69,70]. There remain no examples of differentially-methylated regions that are causally linked to sex differences in gene expression. To date, the best example of the epigenetic effects of perinatal estradiol comes from the gene encoding ERa (*Esr1*). *Esr1* is expressed in the brains of both sexes beginning in mid-gestation [71] and is downregulated by perinatal estradiol via an unknown mechanism [72], leading to increased expression of this receptor in some brain areas in females compared to males [73,74]. 

There are many reports of sex differences in DNA methylation of the *Esr1* promoter [66,67,68,75,76,77,78,79], although the residues identified as differentially hypo- or hyper-methylated vary across studies. In humans, the proximal promoter of human *ESR1* is hypomethylated across tissues, suggesting that variation in methylation at individual promoter CpGs is not causal for expression levels of *Esr1*. In fact, all CpGs at promoters are hypomethylated across the genome, regardless of the level of expression of the associated gene [64]. However, methylation at upstream alternative *Esr1* promoters is tissue-specific, consistent with work demonstrating that *Esr1* is expressed from these alternate promoters in distinct tissues and tumor types [68,76,80,81]. WGBS across multiple developmental time points could reveal distal regulatory elements that maintain sex differences in the expression of *Esr1* and other estrogen-regulated genes [82]. Yet it is still unclear how estrogens would directly alter DNA methylation. There is little evidence of interaction between ERα and DNMT or Tet enzymes, although both DNA methylation and loss of TET2 can attenuate gene activation by ERα in breast cancer cell lines and tumors [83,84,85]. 

### 3.2. Histone Modifications

In addition to DNA methylation, chemical modification of amino acid residues primarily within the N-terminal tail of histone proteins has long been associated with epigenetic regulation of gene expression [86]. Such modifications physically alter chromatin structure via electrostatic interactions as well as attract reader proteins that exert diverse functions, such as chromatin remodeling and recruitment of transcriptional machinery [87]. Because certain histone PTMs, either alone or in combination, are strongly associated with distinct classes of cis-regulatory elements (“histone code” hypothesis) and can persist in the absence of an initiating signal, they are often considered an epigenetic modification [86,88,89].

The perinatal hormone surge has been hypothesized to impart such histone PTMs, resulting in chromatin states that maintain sex-specific gene expression programs [38,90]. Although such chromatin states have not been described, there is an extensive characterization of the interaction of ERα with histone acetyltransferases (HATs), methyltransferases, and deacetylases (HDACs), the SWI/SNF nucleosome remodeling complex, and Mediator protein [91]. ERα is tightly associated with steroid receptor coactivators (SRCs), the p160 coactivator, and p300, which has HAT activity and occupies active enhancers [12,92,93]. Broad manipulation of coregulator or HDAC function disrupts brain development [94,95,96,97], but as with DNA methylation, there are few examples of persistent histone modifications or transcription factor occupancy at specific loci [43]. One study evaluated sex differences in trimethylation of lysine 4 on histone H3, a histone modification found at the promoters of active genes, but found little correlation between increased promoter H3K4me3 and gene expression [98].

One common criticism of the role of histone PTMs in epigenetic maintenance is that active chromatin marks are thought to depend on transcriptional activators for renewal [2,99]. In the absence of an epigenetic initiator, histone acetylation has a comparatively short half-life on core histones [100], although H3K4me1 persists longer and is present on poised genes that are transcribed at low levels [101,102,103]. In contrast, histone PTMs associated with repressed chromatin, such as H3K27me3 and H3K9me3, can be generated and recognized by the same protein complex, constituting an intrinsic positive feedback loop. The Polycomb group proteins, which assemble into polycomb repressive complex 1 (PRC1) or 2 (PRC2), fulfill this dual reader-writer function and play well-known roles in stem cell differentiation, as well as cell-type maintenance [104,105]. The involvement of PRC1/2 in maintaining neuron cell fate is an active area of investigation [106,107,108,109]. Although ERα has primarily been shown to recruit co-activator complexes upon ligand binding, it can also recruit co-repressors that are expressed in the brain [91,110]. Thus, neonatal estradiol could impart self-sustaining repressive chromatin in males, resulting in a female-bias in the expression of certain genes or a female-specific transcriptional response to a later signaling cue. 

### 3.3. Genome Organization

Advances in imaging and genomic technologies have made it possible to observe the 3D structure of the genome, revealing that interphase chromosomes are organized into distinct compartments so that active genes are located in the same physical space within the nucleus [111,112]. It is now thought that genome organization itself constitutes an additional level of epigenetic regulation as differentiated cells exhibit unique patterns of chromosomal compartmentalization. Genome organization through interaction with nuclear architecture appears to be of particular importance in neurons, which must maintain a defined cell identity program throughout the life of the organism while retaining the capacity for rapid transcriptional responses to neural activity [113,114,115]. Alterations to nuclear organization can lead to low-level dysregulation of many genes and have been suggested to underlie neurological and psychiatric disorders, as mutations in many nuclear architecture proteins are associated with disease [114,116,117,118,119,120]. 

Similar to other transcription factors, nuclear hormone receptors regulate gene expression by looping distal enhancer elements and associated regulatory machinery to their target promoters [121,122,123,124,125,126,127]. As hormones can induce the expression of thousands of genes, large-scale rearrangements of chromatin can occur, resulting in significant structural changes in the genome [128,129]. Recent analysis of genome organization in breast cancer cells has revealed large-scale coordinated transcriptional responses to ligand stimulation [130]. Steroid-responsive genes often cluster in groups that are jointly activated or repressed by hormone receptors [122,128,130,131,132]. The acute effects of estradiol on nuclear organization in the brain were first demonstrated in a pioneering electron microscopy study of the ventromedial hypothalamus, which contains many ERα-expressing neurons [133]. The authors implanted gonadectomized female rats with estradiol or sham Silastic capsules and assessed ultrastructural and morphometric nuclear changes. Remarkably, they found that 2 hours of estradiol exposure was sufficient to initiate a robust transcriptional response with significant changes in the size and shape of nuclei, a loss of nuclear envelope invaginations and a decrease in clumped heterochromatin in the nucleoplasm [133]. Taken together, the results from this classic study in the brain combined with more recent work on coordinated gene regulation by ERα in cell lines suggest steroid hormones can “organize” the developing brain by changing the 3D structure of the genome. 

## 4. Sexual Differentiation of the Brain is Developmental Programming

How might sex differences in the epigenome arise throughout development? The simplest and perhaps oldest example of epigenetic regulation is a self-perpetuating transcriptional response that persists in the absence of the original signal [2,134]. When considering sexual differentiation of the brain, it is assumed that sex differences in brain function occur solely from the alteration of the epigenome via covalent modification of DNA or histones. However, cell fate changes are often regulated by the induction of a transcription factor that can then auto-regulate its own expression and establish new cell-type-specific gene expression programs or repress genes associated with alternate fates [135,136,137,138]. In neurodevelopment, this logic has been most clearly elucidated in *C. elegans*, in which terminal selector transcription factors activate and maintain suites of genes that define differentiated neuron types [139,140].

**Figure 2:**
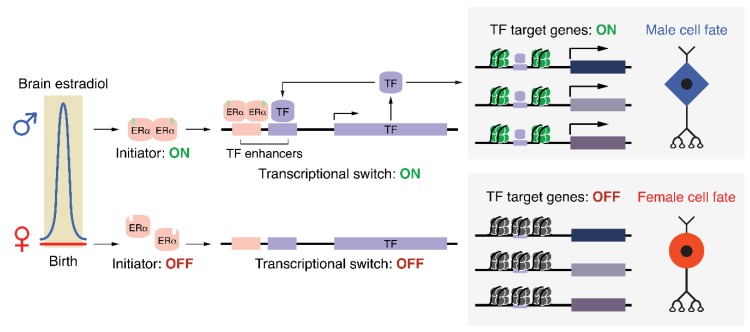
Proposed positive feedback mechanism responsible for maintaining sex-specific cell fates. ERα: estrogen receptor alpha; TF: transcription factor.

We propose that developmental hormone signaling directs sexual differentiation of the brain by initiating a positive feedback mechanism that specifies a male-typical cell fate (Figure 2). This process could occur by direct activation of a lineage-defining factor by nuclear hormone receptors, or by the cooperation of these receptors with other transcription factors, as shown in other tissues. Cell-type-specific repertoires of transcription factors and cofactors could then activate or repress distinct cohorts of genes in response to a broad hormone stimulus. Such programs may include genes involved in neurite extension, energy metabolism, synapse formation, or cell adhesion, leading to sex differences in neurodevelopmental trajectories. Importantly, once bound to cis-regulatory elements, lineage-defining factors would recruit cofactors that enzymatically modify chromatin, producing an epigenomic signature that persists in the absence of the original initiating estradiol signal. Although such a signature reflects the activation status of its target genes, it alone does not reveal the epigenetic process responsible for establishing and/or maintaining that gene’s expression pattern.

One possible mechanism by which perinatal ERα activation could produce a sex difference in cell fate, as shown thematically in Figure 1. In males (*upper*), brain estradiol activates a transcriptional initiator (ERα), which turns on the expression of one, or potentially several, transcription factors (TFs). These TFs act as an irreversible ‘transcriptional switch’ by maintaining their expression via positive feedback while also activating genes responsible for producing a male cell fate. Importantly, despite the initiator returning to an inactive state shortly after birth, the ‘transcriptional switch’ remains active, fulfilling Waddington’s definition of an epigenetic process. In females (*lower*), the lack of estradiol during the perinatal window results in the ‘transcriptional switch’ never being turned on, which, due to the lack of induction of TF target genes, produces the default female cell fate. Epigenomic modifications associated with active or poised genes are schematized as green nucleosomes in males. These same genes in females would reside in a repressed or silenced chromatin state (black nucleosomes).

A conceptual precedent for long-term gene regulation in response to transient hormonal signaling has been observed in *D. melanogaster*. In this species, the hormone ecdysone, which is released in pulses throughout development, controls the transition from early- to late-born neurons within mushroom bodies (MBs) – structures that process olfactory information. In early-born MB neurons, the transcription factor *Chinmo* maintains the expression of the ecdysone receptor (EcR-B1). During the larval-pupal transition, a surge of ecdysone activates EcR-B1, which signals for the persistent downregulation of *Chinmo* and irreversible transition to late-born MB neurons [141]. A similar mechanism occurs in mid-larval neuroblasts, which undergo temporal changes in transcription factor expression to produce different neural cell fates [142]. Indeed, the intersection of steroid hormone release with brain developmental stage varies across species: The critical period for sexual differentiation of the brain can be determined by assessing adult behavioral responses to hormone treatment at different time points. In altricial species that are less developed at birth, the critical period extends into postnatal life, whereas in animals that are more mature at birth, including humans, the critical period occurs during fetal development [143,144]. 

## 5. Sex Differences in Gene Regulation May Underlie Sex Differences in Disease Susceptibility

Humans also experience a testosterone surge just after birth, although the role of neonatal hormones in sexual differentiation of the human brain is not known. Rather, a sustained rise in fetal testosterone level during gestational weeks 8–20 is thought to direct masculinization of the brain. Girls with congenital adrenal hyperplasia (CAH) have elevated fetal testosterone and show male-typical levels of childhood aggression, play behaviors, and spatial skills [145,146,147,148]. Throughout the fetal testosterone surge, the brain undergoes extensive neurogenesis and neuronal migration. Although the cell populations expressing hormone receptors and aromatase in the developing human brain have not been identified, it is intriguing to speculate that early testosterone exposure in boys alters the epigenome or chromatin organization of AR target loci, leading to increased variability in gene expression, brain development, and behavior. Identification of hormone-regulated gene programs in the developing brain is likely to reveal sex-specific vulnerabilities: If a neuronal population expresses more of a given gene in females compared to males, females may be less susceptible to heterozygous loss-of-function of that gene. Moreover, genetic variants within non-coding regulatory elements that bind hormone receptors could affect disease susceptibility in a sex-specific manner. A female-specific genetic vulnerability may also result from fluctuating gene expression over the course of the estrous cycle, such a vulnerability could be unmasked by puberty or menopause, both of which are times of increased diagnoses of mental health conditions in women [149,150]. 

Many psychiatric disorders show a sex bias in incidence and etiology [45,151,152]. A current goal for researchers is to determine whether sex differences in gene expression or regulation can contribute to widely-documented sex differences in disease. Certainly, there are many conditions in which mutations in X-linked genes cause more severe phenotypes in males, which lack an additional functional copy of the gene. However, to the best of our knowledge, there are no examples of sex differences in expression of a particular gene contributing to a corresponding difference in disease incidence or progression. Autism spectrum disorders (ASD) are diagnosed more frequently in boys than girls (4:1), and there is substantial evidence that a genetic component drives this sex-bias [153]. Significant advances in generating functional genomic data from human brains, including ChIP-seq, bisulfite sequencing, and Hi-C, has been transformative for the field of psychiatric genetics. These data allow specific loci to be correlated with gene expression, enhancer activity, and chromatin organization, thereby linking target genes to genetic variants identified in GWAS studies of psychiatric disorders [154,155,156,157,158,159,160,161]. Although no sex differences in regulatory elements have been identified in these studies, they largely focus on cortical areas in which few sex differences in gene expression have been reported in rodents [17]. Interestingly, genes with *de novo* post-zygotic mutations in individuals with ASD are highly expressed in the fetal amygdala, a region rich in hormone receptors [162]. More extensive epigenomic analysis of human subcortical brain regions that display sex differences in corresponding rodent areas may reveal conserved hormone-regulated gene programs. In the final section of this review, we will discuss experimental approaches and technical innovations that can be applied in mice to identify the transcriptional events that direct sexual differentiation of the brain.

## 6. Towards a *bona fide* Epigenetic Mechanism Underlying Sex Differences in the Brain

Do epigenomic sex differences reflect persistent effects of ERs themselves? Or are they a signature of developmental events that occur in one sex but not the other, as a consequence of ER target genes which are no longer active? In order to distinguish between these possibilities, it is necessary to determine the genomic targets of estradiol signaling at birth and to monitor gene expression and chromatin in females, males, and females given estradiol at birth throughout postnatal development. Genes that show increased expression in males and estradiol-treated females at birth may be transient but necessary for masculinization. Similarly, the histone modification H3K27Ac marks active enhancers that will change over time as differentiation and brain wiring progresses. Such experiments would also reveal whether sex differences in gene expression in adulthood are a direct consequence of neonatal or pubertal estradiol signaling or occur via hormone-independent mechanisms.

Studies of gene regulatory mechanisms in the brain have suffered from the same challenges faced by neuroscience in general: How does one achieve spatial and temporal specificity in a highly heterogeneous tissue using mouse genetic tools? Most efforts have employed broad genetic deletion of chromatin modifiers or even pharmacological approaches, which inhibit all histone deacetylation or DNA methylation, to demonstrate the necessity of an individual epigenomic modification. Such brute force approaches will misregulate many genes, not just the one or few of interest, confounding interpretation about the role of an individual locus. One approach to determine a causal role for a regulatory element is to genetically delete that element or target it with a CRISPR/Cas9 system; tethering histone-modifying domains to a catalytically inactive Cas9 is currently the most promising strategy in the brain [163,164,165]. Enhancers are often highly redundant [166], and several may need to be edited at once to achieve an effect; such an experiment has already been performed for ERα enhancers in breast and endometrial cell lines [167]. 

A common assumption in the study of sex differences is that estrogen-regulated genes contain canonical estrogen receptor response elements (EREs) in their promoters. However, genome-wide studies of ERα binding have revealed that the majority of binding occurs at distal and intronic elements, often via interactions with cell-type-specific TFs [168,169,170]. Accordingly, ERα genomic binding depends on TF co-expression within a particular tissue or cell-type, as shown by comparing RNA-seq and ERα ChIP-seq datasets between mouse liver and aorta, mouse uterus and efferent ductules, and human endometrial and breast cancer cell lines [168,170,171,172]. Such comparisons have implicated broad classes of TF families in context-dependent ERα genomic binding, such as Homeobox (Hox) factors in the uterus [168]. Mass spectrometry-based methods can subsequently identify specific proteins that interact with ERα in different tissues, as done previously in breast cancer cells [173]. To date, however, the genomic binding sites and protein binding partners of ERα have not been examined in brain tissue. 

As ERα is recruited to distinct genomic sites in endometrial tissue compared to mammary tissue, there may also be different estrogen-regulated genes in, for example, cortical parvalbumin neurons compared to hippocampal pyramidal neurons or astrocytes. The use of the INTACT (Isolation of Nuclei Tagged in Specific Cell Types) genetic labeling system permits the purification of nuclei from Cre-expressing cells, followed by an assessment of histone modifications or DNA methylation by ChIP-seq or WGBS [65,174]. As sex differences in autosomal gene expression are subtle, the use of INTACT and single-cell methods will facilitate the identification of sex differences in neuronal epigenomes by increasing specificity. Although the enzymes that modify DNA and chromatin are largely ubiquitous, their recruitment to individual regulatory elements often depends on sequence-specific transcription factors. Assessment of transcription factor binding by ChIP-seq in discrete brain regions has been stymied by the high number of cells required for the method. Such experiments are now feasible with the introduction of CUT&RUN, which relies on low cell numbers, potentially even single cells, to identify genomic sites of histone modifications or TF binding [175,176]. Ultimately, it should be possible to determine the genomic binding sites of hormone receptors and other transcription factors in defined neuronal populations. Application of the technical advances described above is likely to reveal the dynamics and mechanisms of the gene regulatory mechanisms that coordinate sexual differentiation of the brain or other critical periods in brain development.
